# Reduced Rates of Post-Transplant Recurrent Hepatocellular Carcinoma in Non-Alcoholic Steatohepatitis: A Propensity Score Matched Analysis

**DOI:** 10.3389/ti.2022.10175

**Published:** 2022-07-05

**Authors:** Ryan Lamm, Peter J. Altshuler, Keyur Patel, Osama Shaheen, Angel Paulo Amante, Jesse Civan, Warren Maley, Adam Frank, Carlo Ramirez, Jaime Glorioso, Ashesh Shah, Hien Dang, Adam S. Bodzin

**Affiliations:** ^1^ Department of Surgery, Thomas Jefferson University Hospital, Jefferson University Hospitals, Philadelphia, PA, United States; ^2^ Department of Gastroenterology, Thomas Jefferson University Hospital, Jefferson University Hospitals, Philadelphia, PA, United States

**Keywords:** United Network for Organ Sharing, hepatocellular carcinoma, non-alcoholic steatohepatitis, recurrence, Organ Procurement and Transplantation Network

## Abstract

Non-alcoholic steatohepatitis (NASH)-related hepatocellular carcinoma (HCC) has become the second leading cause of HCC-related liver transplantation in the United States. This study investigated post-transplant recurrence and survival for patients transplanted for NASH-related HCC compared to non-NASH HCC etiologies. Retrospective review of the United Network for Organ Sharing (UNOS) Organ Procurement and Transplantation Network (OPTN) database identified 7,461 patients with HCC—1,405 with underlying NASH and 6,086 with non-NASH underlying diseases. After propensity score matching (PSM) to account for patient- and tumor-related confounders 1,175 remained in each group. Primary outcomes assessed were recurrence rate and recurrence-free survival. Recurrent malignancy at 5 years post-transplant was lower in NASH compared to non-NASH patients (5.80 vs. 9.41%, *p* = 0.01). Recurrence-free survival, however, was similar at 5 years between groups. Patients with NASH-related HCC were less likely to have post-transplant recurrence than their non-NASH counterparts, although recurrence-free survival was similar at 5 years.

## Graphical Abstract

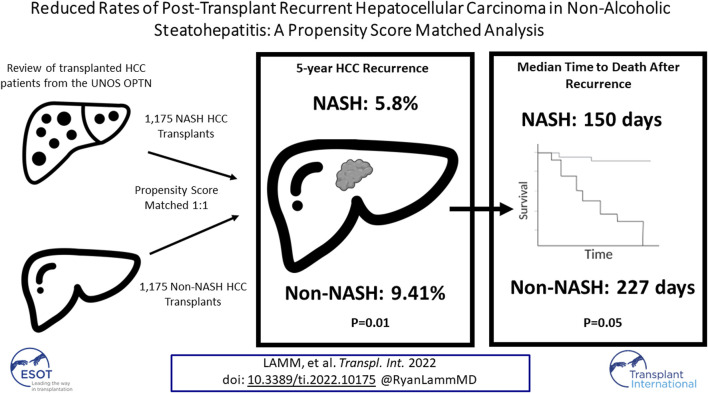

## Introduction

Hepatocellular carcinoma (HCC) accounts for the fourth most cancer-related deaths in the United States (US) (1). Despite a recent national decline in the incidence of HCC cases, HCC secondary to non-alcoholic steatohepatitis (NASH) has become the fastest growing cause of HCC amongst liver transplant registrants in the US (2). This correlates to the increased rates of transplantation for NASH, currently representing the most common indication for liver transplantation in females and the second most common overall (3). As the obesity epidemic continues, it is becoming increasingly important to understand the outcomes associated with this subset of the HCC cohort.

HCC develops through progressive hepatocellular inflammation, leading to fibrosis, cell death, and aberrant regeneration which results in tumor formation (4). Different underlying etiologies uniquely impact gene regulation and cellular function leading to disease progression (4). World-wide, viral hepatitides (hepatitis C virus [HCV] and hepatitis B virus [HBV]) remain the most frequent etiologies of HCC; however, in the United States the burden of viral hepatitis-related HCC has been reduced by preventative treatment including the HBV vaccine and direct-acting antiviral (DAA) therapies for HCV (5–7). In contrast to viral hepatitis, as the obesity epidemic and prevalence of metabolic syndrome increases, non-alcoholic fatty liver disease (NAFLD) has become a progressively more common cause of end-stage liver disease (ELSD) (8). NAFLD currently afflicts 25% of the US population, with 20% of these patients demonstrating hepatocellular ballooning, inflammation, and steatohepatitis characteristic of NASH (9, 10).

Owing to the underlying metabolic syndrome often associated with NASH, these patients carry higher rates of concomitant cardiovascular and endocrine comorbidities than non-NASH ESLD population (11). Despite this, previous studies evaluating transplantation for NASH have consistently demonstrated similar post-transplant outcomes compared to patients with non-NASH liver failure (11, 12). Few studies, however, have assessed transplantation for NASH-related HCC which has increased in prevalence every year since 2002 (13). Specifically, little is known regarding recurrence rates and post-transplant survival in these patients compared to their non-NASH counterparts. This study sought to assess post-transplant recurrence rates and survival for NASH compared to non-NASH populations, as well as investigate survival patterns in patients with recurrent HCC after transplant.

## Methods and Patients

### Patient Population

**FIGURE 1:**
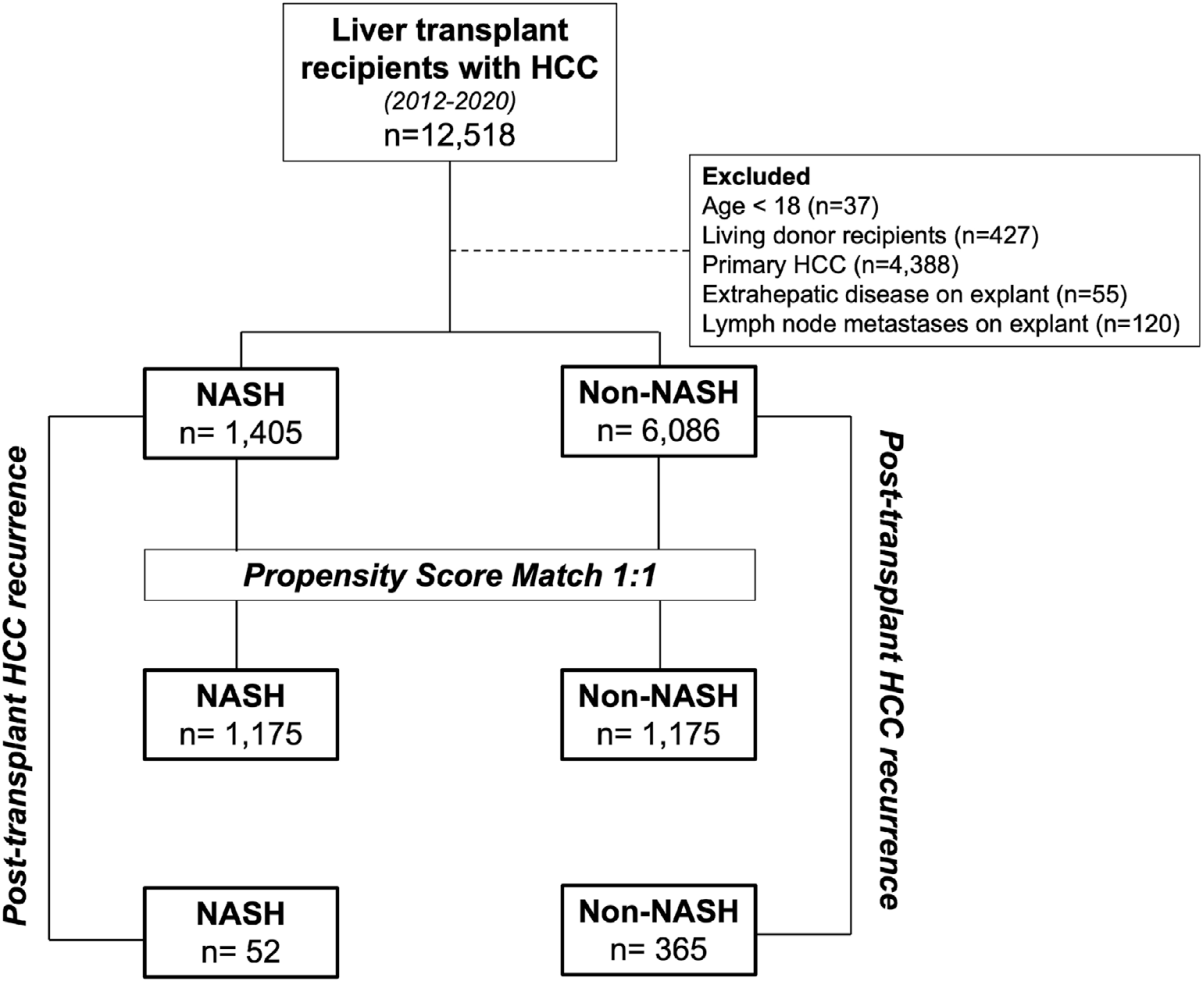
Study design. NASH transplant recipients with HCC were first compared to non-NASH recipients with HCC. These patients were then propensity matched and further compared. Additional analysis was performed on the unmatched populations to compare with post-transplant HCC recurrence and post-recurrence survival between NASH and non-NASH populations.

We performed a retrospective review of the Organ Procurement and Transplantation (OPTN) database for all adult (≥18-year-old) deceased donor liver transplant recipients in the United States diagnosed with HCC in the setting of known underlying liver disease. Our study population included transplants from 4 November 2012 to 6 December 2020, with the initiation date coinciding to the date OPTN began tracking tumor characteristics on transplant hepatectomy specimens. Recipients were first classified by diagnosis of NASH (NASH: 1,405, non-NASH: 6,086; Figure 1). Non-NASH patients with a primary HCC and no precipitating liver disease (i.e., HCV, alcoholic cirrhosis, HBV) were excluded, as were those with evidence of extrahepatic spread or lymph node metastases on explant. To account for the high rate of undiagnosed NASH in patients with cryptogenic cirrhosis (14, 15), those with cryptogenic cirrhosis and underlying diabetes or BMI ≥30 were included in the NASH population, consistent with the methodology of previously validated, published studies (16–18). Patients were then stratified by post-transplant HCC recurrence, with cases of recurrent HCC identified through malignancy follow-up data (19). Here, NASH and non-NASH populations with recurrent malignancy were compared (NASH: 52, non-NASH: 365). Approval to conduct this analysis was obtained from the Thomas Jefferson University Institutional Review Board.

### Assessing Post-Transplant Hepatocellular Carcinoma Recurrence Rate in NASH and Non-NASH Recipients

We first set out to assess post-transplant HCC recurrence rate in NASH vs non-NASH patients. We defined recurrence rate as a post-transplant HCC-related death or a diagnosis of HCC recurrence, derived from a validation study showing reliability of HCC recurrence data in the UNOS OPTN database (19). To reduce confounding bias associated with recipient cohorts of interest, non-NASH patients were propensity score matched (PSM) to NASH patients (Supplementary Figure S1). Both unmatched and PSM cohorts were compared with respect to baseline recipient, donor, and transplant characteristics. Tumor characteristics on transplant hepatectomy were also compared.

As most cases of recurrent HCC occur within 5 years (20), primary analysis focused on 5-year post-transplant recurrence rates. Secondary outcomes included median time to recurrence for those with recurrent HCC following transplant, and overall survival in NASH and non-NASH patients.

### Evaluating Survival After Post-Transplant Recurrence

We then assessed survival patterns in NASH and non-NASH patients who developed post-transplant recurrence. Here, patients with recurrent HCC after transplant were again divided by underlying diagnosis (NASH: 52, non-NASH: 365). Baseline recipient, donor, and transplant characteristics were compared, as were tumor characteristics on transplant hepatectomy. The primary outcome assessed was survival after recurrence.

To evaluate differences between NASH and non-NASH patient cohorts’ overall survival after transplant with and without recurrence, and to verify any trends seen only in the recurrence population, overall survival was reported in all four of those subgroups.

### Statistical Analysis

Continuous variables were evaluated for normality using the Shapiro Wilk test. Non-normally distributed variables were compared with a Wilcoxon rank-sum test and were represented as median interquartile range (IQR). Categorical variables were compared using a chi-square or Fisher’s exact test and were represented as numbers (percentage of population).

PSM of non-NASH to NASH patients was completed using 1:1 nearest-neighbor matching with a caliper width of 0.2. Covariates matched in propensity score models were identified *a priori* or by regression analysis as recipient, tumor explant, and donor characteristics predictive of graft survival. Appropriate matching was confirmed through histogram analysis of propensity score distributions and by Rubin’s Bias and Ratio tests comparing matched cohorts. Full details regarding the PSM, including covariates used in the match, can be found in Supplementary Figure S1.

Post-transplant HCC recurrence rates were assessed using a competing risk-regression model with non-cancer-related death used as a competing outcome. Cumulative incidence of HCC recurrence was evaluated using Fine-Gray proportional sub distribution hazard ratio (SHR) models in NASH and non-NASH recipients. Post-transplant survival and survival after diagnosis of recurrence, as defined above, were reported via Kaplan-Meier curves with statistical significance assessed using Log-rank tests. Recurrence rates were compared using Cox Proportional Hazard regression modeling. These data were remained unadjusted as attempts at adjusted analyses yielded underpowered results. `For all comparisons two-sided statistical significance was set *a priori* at *p < 0.05*. All statistical analyses were performed using Stata/MP 16.1 (Statacorp, College Station, TX).

## Results

### Post-Transplant Recurrence Rates in NASH and Non-NASH Patients

#### Baseline Characteristics of Hepatocellular Carcinoma Patients by Diagnosis of NASH

Prior to propensity matching, 1,405 patients had NASH-related HCC compared to 6,086 with non-NASH diagnoses (Supplementary Tables S1, S2). Median follow-up was 924 days (IQR: 365–1,707) in the NASH cohort and 1,366 days (IQR 678–1,898) for the non-NASH cohort. Underlying diseases in the non-NASH population were as follows: HCV (66.44%), HBV (6.34%), EtOH (21.30%) and “Other,” which included metabolic, cholestatic and autoimmune conditions (5.92%).

PSM resulted in 1,175 matched pairs with largely similar profiles (Tables 1, 2). Median follow-up was 1,070 days for NASH (IQR: 382–1,809) and 1,243 days (IQR 668–1,903) for non-NASH. In the PSM non-NASH group, HCV was the underlying diagnosis in 65.15% of patients (*n* = 759), while 5.41% (*n* = 63) had HBV, 21.55% (*n* = 251) EtOH and 7.90% (*n* = 92) other. No significant differences were observed in recipient or transplant profiles, or in tumor explant characteristics.

**TABLE 1:** Propensity score matched baseline characteristics between NASH and non-NASH recipients with HCC.

	NASH	Non-NASH	*p*-value
Number	1,175	1,175	
Median followup (days)	1,070 (382–1,809)	1,243 (688–1,903)	
Recipient characteristics			
Age	64 (60–68)	64 (60–67)	0.55
Female sex	378 (32.17%)	384 (32.68%)	0.83
Ethnicity			0.67
White	882 (75.06%)	865 (73.62%)	
Black	11 (0.94%)	14 (1.19%)	
Other	282 (24.00%)	296 (25.19%)	
BMI	31.79 (28.20–35.53)	27.75 (24.64–31.66)	<0.01
Pre-exception MELD	12 (9–16)	12 (9–16)	0.48
AFP			0.98
<100 ng/ml	1,097 (93.36%)	1,094 (93.11%)	
100–399 ng/ml	62 (5.28%)	65 (5.53%)	
≥400 ng/ml	16 (1.36%)	16 (1.36%)	
Locoregional therapy			
TACE	752 (64.00%)	759 (64.60%)	0.79
TARE	132 (11.23%)	140 (11.91%)	0.65
Ablation	384 (32.68%)	365 (31.06%)	0.43
Other	11 (0.94%)	13 (1.11%)	0.84
Number of locoregional treatments			0.69
0	126 (10.72%)	116 (9.87%)	
1	727 (61.87%)	747 (63.57%)	
2	254 (21.62%)	238 (20.26%)	
≥3	68 (5.79%)	74 (6.30%)	
Disabled functional status	165 (14.04%)	184 (15.66%)	0.29
Diabetes mellitus	818 (71.57%)	328 (28.20%)	<0.01
Portal vein thrombosis	189 (16.11%)	203 (17.32%)	0.44
Hemodialysis	10 (0.85%)	19 (1.62%)	0.13
Previous abdominal surgery	626 (53.28%)	610 (51.91%)	0.53
Multiorgan	20 (1.70%)	23 (1.96%)	0.76
Primary diagnosis			—
NASH	1,175 (100.00%)	0 (0.0%)	
HCV	0 (0.0%)	63 (5.41%)	
HBV	0 (0.0%)	759 (65.15%)	
EtOH	0 (0.0%)	251 (21.55%)	
Other^a^	0 (0.0%)	92 (7.90%)	
Donor characteristics			
Age	46 (30–58)	45 (31–59)	0.80
Female sex	492 (41.87%)	499 (42.47%)	0.80
BMI	27.46 (23.74–32.34)	27.65 (23.56–31.96)	0.76
Diabetes mellitus	159 (13.53%)	165 (14.04%)	0.76
Macrosteatosis (%)	5 (0–10)	5 (0–10)	0.08
Inotrope support	566 (48.17%)	556 (47.32%)	0.71
LDRI	1.58 (1.28–1.92)	1.60 (1.28–1.94)	0.22
Cause of death			0.36
Anoxia	420 (35.74%)	459 (39.06%)	
CVA	391 (33.28%)	391 (33.28%)	
Head trauma	337 (28.68%)	302 (25.70%)	
CNS tumor	8 (0.68%)	5 (0.43%)	
Other	19 (1.62%)	18 (1.53%)	
DCD	84 (7.15%)	83 (7.06%)	0.99
Transplant details			
CIT (hours)	5.90 (4.60–7.25)	5.93 (4.50–7.55)	0.43

Values are listed as number (percentage) or median ± interquartile range unless otherwise stated. BMI, body mass index; NASH, non-alcoholic steatohepatitis; AFP, alpha fetoprotein; TACE, transarterial chemoembolization; TARE, transarterial radioembolization; HCV, Hepatitis C Virus; EtOH, alcohol; CVA, cerebrovascular accident; LDRI, Liver Donor Risk Index; CNS, central nervous system; DCD, donation after cardiac death; CIT, cold ischemia time.

a Includes metabolic, autoimmune and cholestatic diseases.

**TABLE 2:** Propensity score matched tumor characteristics in transplant hepatectomy specimens.

	NASH	Non-NASH	*p*-value
Number	1,175	1,175	
No tumor on explant	71 (6.04%)	76 (6.47%)	0.73
Number of tumors			0.83
1	548 (46.64%)	524 (44.60%)	
2	268 (22.81%)	269 (22.89%)	
3	128 (10.89%)	130 (11.06%)	
≥4	160 (13.62%)	176 (14.98%)	
Largest tumor size (cm)	2.5 (1.5–3.5)	2.4 (1.5–3.5)	0.59
Tumor differentiation^a^			0.75
Complete necrosis	296 (25.19%)	276 (23.49%)	
Well	274 (23.32%)	270 (22.98%)	
Moderate	532 (45.28%)	555 (47.23%)	
Poor	73 (6.21%)	74 (6.30%)	
Vascular invasion			0.86
Microvascular	125 (10.64%)	134 (11.40%)	
Macrovascular	21 (1.79%)	21 (1.79%)	
Satellite lesions	59 (5.02%)	61 (5.19%)	0.93

Values are listed as number (percentage) or median ± interquartile range unless otherwise stated.

a Differentiation of worst tumor.

#### Outcomes of Hepatocellular Carcinoma Patients by Diagnosis of NASH

**FIGURE 2:**
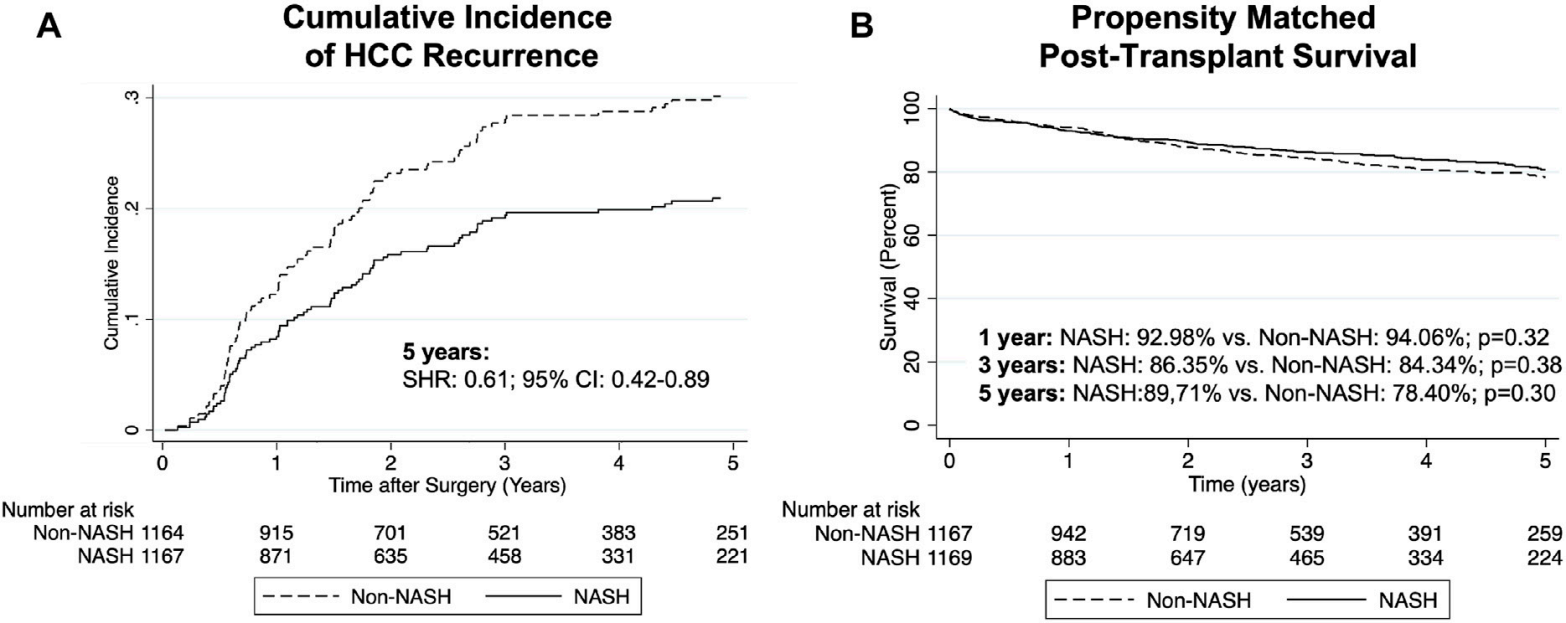
Cumulative incidence of post-transplant HCC recurrence **(A)** and Kaplan-Meier curves comparing survival **(B)** in NASH vs PSM non-NASH patients.

Comparing NASH to non-NASH transplant recipients, we observed reduced post-transplant HCC recurrence rate in NASH patients. After PSM, recurrence rates at 5 years were 5.80% in the NASH group and 9.41% in non-NASH patients (SHR: 0.61, 95% CI: 0.42–0.89, *p* = 0.01; Table 3 and Figure 2A). For patients with post-transplant HCC recurrence, however, we could not show significant differences between median time to recurrence (426 vs. 400 days, *p* = 0.59). Additionally, while recurrent rates were reduced in NASH patients, overall survival was not statistically significantly different (HR: 0.87, 95% CI: 0.71–1.07, *p* = 0.20, Figure 2B). At 1 year, survival in NASH patients was 92.98% and in non-NASH patients 94.06% (*p* = 0.32); at 3 years, survival was 86.35% vs. 84.34% (*p* = 0.38), and at 5 years, 80.71% vs. 78.40% (*p* = 0.30), thus all non-significant.

**TABLE 3:** Propensity score matched transplant outcomes by diagnosis of NASH.

	NASH	Non-NASH	HR/SHR	95% CI	*p*-value
Number	1,175	1,175			
Acute Rejection within 1 year	77 (8.85%)	62 (7.17%)	—	—	0.78
Recurrent Malignancy			(SHR)		
5-year	5.80%	9.41%	0.61	0.42–0.89	0.01
Median time to recurrence^a^	426 (213–752)	400 (195–796)	—	—	0.59
Post-transplant survival			(HR)		
Overall	—	—	0.87	0.71–1.07	0.20
1-year	92.98%	94.06%	—	—	0.32
3-year	86.35%	84.34%	—	—	0.38
5-year	80.71%	78.40%	—	—	0.30

Values are listed as percent, number (percentage) or median ± interquartile range unless otherwise stated.

a For patients with recurrent HCC only.

### Assessing Survival Following Post-Transplant HCC Recurrence in NASH and Non-NASH Populations

#### Baseline Characteristics of Patients With Recurrent Hepatocellular Carcinoma by Diagnosis of NASH

We next assessed only patients with recurrent HCC after transplant. In this cohort, median follow-up for NASH patients was 2,059 days (IQR: 1,003–2,157) and 2,132 days (IQR: 1,445–2,409) for non-NASH patients. As shown in Table 4, we found that NASH patients were older (65 vs. 61 years old, *p* < 0.01), more frequently female (36.54% vs. 17.53%, *p* < 0.01), and comprised different ethnicities. Again, they also carried higher BMI (32.39 vs. 27.40, *p* < 0.01) along with increased incidence of diabetes (62.00% vs. 26.52%, *p* < 0.01) and PVT (25.00% vs. 12.36%, *p* = 0.02). No significant differences were noted in pre-transplant locoregional therapies, donor characteristics or transplant details. Additionally, tumor explant characteristics, were similar between NASH and non-NASH patients with recurrent HCC (Table 5).

**TABLE 4:** Baseline characteristics in NASH and non-NASH recipients with HCC recurrence after transplant.

	NASH	Non-NASH	*p*-value
Patients with recurrent HCC	52	365	
Median followup (days)	2,058 (1,002–2,156)	2,133 (1,444–2,503)	
Recipient characteristics			
Age	65 (62–67)	61 (57–65)	<0.01
Female sex	19 (36.54%)	64 (17.53%)	<0.01
Ethnicity			0.01
White	37 (71.15%)	232 (63.56%)	
Black	0 (0.0%)	50 (13.70%)	
Other	15 (28.85%)	83 (22.74%)	
BMI	32.39 (29.21–35.39)	27.40 (24.27–31.32)	<0.01
Pre-exception MELD	12 (9–16)	11 (8–15)	0.66
AFP			0.65
<100 ng/ml	41 (80.39%)	271 (75.70%)	
100-399 ng/ml	6 (11.76%)	61 (17.04%)	
≥400 ng/ml	4 (7.84%)	26 (7.26%)	
Locoregional therapy			
TACE	38 (73.08%)	264 (72.33%)	0.99
TARE	6 (11.54%)	24 (6.58%)	0.24
Ablation	17 (32.69%)	98 (26.85%)	0.41
Other	0 (0.00%)	4 (1.10%)	0.99
Number of locoregional treatments			0.99
0	6 (11.54%)	41 (11.24%)	
1	27 (51.92%)	194 (53.15%)	
2	14 (26.92%)	94 (25.75%)	
≥3	5 (9.62%)	36 (9.86%)	
Disabled functional status	6 (11.54%)	60 (16.44%)	0.42
Diabetes mellitus	31 (62.00%)	96 (26.52%)	<0.01
Portal vein thrombus	13 (25.00%)	45 (12.36%)	0.02
Hemodialysis	0 (0.00%)	6 (1.64%)	0.99
Previous abdominal surgery	22 (42.31%)	154 (42.19%)	0.99
Multiorgan recipient	0 (0.00%)	7 (1.92%)	0.60
Primary diagnosis			—
NASH	52 (100.00%)	0 (0.0%)	
HCV	0 (0.0%)	245 (67.68%)	
HBV	0 (0.0%)	18 (4.97%)	
EtOH	0 (0.0%)	83 (22.93%)	
Other^a^	0 (0.0%)	16 (4.42%)	
Donor characteristics			
Age	42 (26−56)	44 (30−56)	0.73
Female sex	22 (42.31%)	151 (41.37%)	0.99
BMI	27.23 (23.99–31.65)	27.27 (23.13–31.44)	0.71
Diabetes mellitus	5 (9.62%)	47 (12.88%)	0.66
Macrosteatosis	5 (5–18)	5 (0–10)	0.06
Inotrope support	26 (50.00%)	182 (49.86%)	0.99
LDRI	1.53 (1.23–1.87)	1.54 (1.27–1.87)	0.83
Cause of death			0.98
Anoxia	20 (38.46%)	132 (36.16%)	
CVA	18 (34.62%)	127 (34.79%)	
Head trauma	14 (26.92%)	100 (27.40%)	
CNS tumor	0 (0.00%)	2 (0.55%)	
Other	0 (0.001%)	4 (1.10%)	
DCD	5 (9.62%)	25 (6.85%)	0.40
Transplant details			
CIT (hours)	6.05 (4.25–8.26)	5.95 (4.66–7.58)	0.58

Values are listed as number (percentage) or median ± interquartile range unless otherwise stated.

BMI, body mass index; NASH, non-alcoholic steatohepatitis; AFP, alpha fetoprotein; TACE, transarterial chemoembolization; TARE, transarterial radioembolization; HCV, Hepatitis C Virus; EtOH, alcohol; CVA, cerebrovascular accident; LDRI, liver donor risk index; CNS, central nervous system; DCD, donation after cardiac death; CIT, cold ischemia time.

a Includes metabolic, autoimmune and cholestatic diseases.

**TABLE 5:** Tumor characteristics in transplant hepatectomy specimens in patients with recurrent HCC after transplant.

	NASH	Non-NASH	*p*-value
Patients with recurrent HCC	52	365	
No tumor on explant	0 (0.00%)	9 (2.47%)	0.61
Number of tumors			0.13
1	21 (40.38%)	138 (37.81%)	
2	8 (15.38%)	80 (21.92%)	
3	11 (21.15%)	35 (9.59%)	
≥4	12 (23.08%)	103 (28.22%)	
Largest tumor size (cm)	3.2 (2.1–4.6)	2.8 (1.7–4.3)	0.09
Tumor differentiation^a^			0.50
Complete necrosis	6 (11.54%)	39 (10.68%)	
Well	4 (7.69%)	48 (13.15%)	
Moderate	29 (53.85%)	207 (56.71%)	
Poor	14 (26.92%)	71 (19.45%)	
Vascular invasion			0.11
Microvascular	12 (23.08%)	113 (30.96%)	
Macrovascular	6 (11.54%)	18 (4.93%)	
Satellite lesions	5 (9.62%)	38 (10.41%)	0.99

Values are listed as number (percentage) or median ± interquartile range unless otherwise stated.

a Differentiation of worst tumor.

#### Outcomes in Patients With Recurrent Hepatocellular Carcinoma by Diagnosis of NASH

**FIGURE 3:**
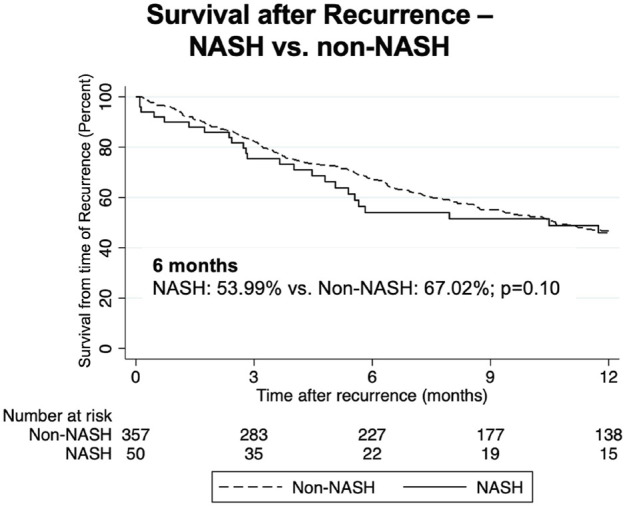
Kaplan-Meier curves comparing survival following recurrence in NASH vs. non-NASH patients.

We then compared outcomes in patients with recurrent malignancy. Here, we found no statistically significant differences in survival from time of recurrence in NASH compared to non-NASH patients (Figure 3; Table 6). At 6 months, survival was 53.99% vs. 67.02, *p* = 0.10; at 1 year, survival was 45.95% vs. 46.71% (*p* = 0.63), and at 18 months 29.03% vs. 34.43% (*p* = 0.45). Further, when measuring median time to death from date of recurrence in those patients with recurrence who had died, time was substantially shorter in NASH patients (150 vs. 227 days, *p* = 0.05), however this finding was not statistically significant (Table 6).

**TABLE 6:** Outcomes in patients with recurrent HCC after transplant by diagnosis of NASH.

	NASH	Non-NASH	HR	95% CI	*p*-value
Patients with recurrent HCC	52	365			
Median time to death after recurrence (days)^a^	150 (73–375)	227 (97–484)	—	—	0.05
Survival after recurrence					
Overall	—	—	1.06	0.73–1.53	0.75
6 months	53.99%	67.02%	—	—	0.10
1 year	45.95%	46.71%	—	—	0.63
18 months	29.03%	34.43%	—	—	0.45

Values are listed as percent, number (percentage) or median ± interquartile range unless otherwise stated.

a For mortalities only.

## Discussion

In this study we compared NASH-related and non-NASH HCC transplant populations, specifically looking at recurrence rates as well as survival post recurrence. NASH patients were found to have a lower HCC recurrence rate at 5 years while post-transplant survival remained similar between the two groups.

Previous studies comparing NASH to non-NASH populations have provided conflicting results to date with regards to HCC outcomes. Billeter et al. utilized propensity-score matching to compare NASH-related and non-NASH HCC patients in 34 NASH patients receiving liver resection in a single institution and found no differences in 1-, 3-, or 5-year recurrence-free survival (21). Furthermore, in a 60 patient cohort, Sadler et. al. noted no difference in overall survival in NASH-related and non-NASH patients receiving liver transplant for HCC (22). Additionally, they observed that meeting Milan criteria did not impact recurrence for NASH-related HCC patients, suggesting that even advanced HCC in NASH may have favorable outcomes (22). While these studies suggested no difference in outcomes for NASH-related HCC, Weinmann et. al. reported decreased overall survival in NASH patients undergoing transplant; however recurrence free survival was not reported (23). Finally, several studies, similarly limited by data on recurrence, have suggested improved overall survival in NASH patients (11, 24, 25). To provide clarity to the conflicting data, our study utilized the largest available national dataset of liver transplant recipients with HCC and found a significantly lower rate of post-transplant HCC recurrence, as well as worse post-recurrence outcomes in the NASH patient population.

Understanding the biology of HCC in NASH-related and non-NASH patients is critical to understanding tumor behavior as well as response to transplantation and adjuvant treatment modalities. Unlike HCC secondary to non-NASH diseases, NASH-related HCC pathogenesis is uniquely affected by a cascade of insulin resistance which causes oxidative stress, inflammation, and fibrosis-stimulating cytokines (4, 26). Additionally, AFP is a frequently used biomarker in screening for HCC associated with tumor aggressiveness since it is produced during times of sustained liver injury and regeneration (27). Studies have found that NASH-related HCC patients have lower levels of AFP and have hypothesized that this may suggest a less aggressive tumor biology (28, 29). Our study similarly noted lower AFP levels in NASH-related HCC patients. Mittal et. al. showed a potential clinical significance of the less aggressive phenotype by noting that NASH-related HCC patients were less likely to be screened for HCC within 3-years of their diagnosis compared to HCV-related, and thus presented at a more advanced stage (28). Despite this, NASH-related HCC patients demonstrated similar 1-year survival to non-NASH patients (28). These findings may help explain the lower recurrence rate we observed in the NASH-related HCC cohort. Ultimately, further studies investigating the biology of post-transplant recurrent HCC and its clinical impact will be critical to define these observations.

Another important difference between NASH-related and non-NASH patients are tumor characteristics at time of surgical treatment. Utilizing the UNOS OPTN database, Lewin et. al. found that NASH patients receiving liver transplantation for HCC were less likely to have tumors with vascular invasion and/or poor differentiation upon explant and were less likely to have evidence of metastasis compared to other HCC etiologies (30). This could support the theory that NASH HCC may be less aggressive at time of surgical intervention, leading to less overall recurrence, but warrants further study.

While we observed lower recurrence rates in NASH HCC patients, those who did recur had shorter median survival than non-NASH patients. Some emerging data may help explain that by highlighting differences in NASH-related HCC response to adjuvant therapies. Locoregional therapy, namely TACE, has been shown to have lower complete response, more progression of disease, higher rates of residual disease, and more recurrence in 1-2-month follow-up imaging in the obese population (31). Wu et. al. attributed this finding to the chronic low level of inflammation associated with obesity which they believed to incite a pro-inflammatory and, thus, tumorigenic metabolic milieu potentially contributing to increased recurrence (31). In addition, resistance to sorafenib, a widely used systemic treatment for late-stage HCC, is observed in patients on chronic metformin therapy as these drugs work on similar downstream pathways (32, 33). Some studies suggest Sorafenib delays time to HCC recurrence and in a small study, Kang et. al. found just over a 7-month survival benefit in a heterogenous population of post-transplant HCC patients with recurrence (34, 35). With a majority of NASH-related HCC patients being obese and having diabetes these findings could provide insight into why we observed that NASH-related HCC patients with recurrence had a significantly shorter survival, although we are limited by the data source. Clearly, further investigation using more a detailed data source is required to explain the recurrent tumor biology associated with the NASH.

Our study suffers several limitations which include but are not limited to the retrospective nature of a large, federally maintained database. It should be noted that HCC outcomes in this database lack granular details regarding some tumor and treatment characteristics. A recent study, however, showed that the UNOS OPTN observed HCC recurrence rate was not significantly lower than the expected rate, validating the use of the OPTN database in evaluating outcomes related to transplantation for HCC (19). Moreover, while we sought to evaluate tumor specific outcomes between NASH-related and non-NASH recipients, we cannot definitively comment on the “biology” of the tumor itself, but can draw attention to the series of comparisons we made between NASH and non-NASH groups of HCC post-transplant patients. As such, future studies should focus their attention on the tumor-specific behavior which contributes to the diversion of these two distinct populations. In addition, our study inclusion period started prior to the widespread use of DAAs, possibly affecting the HCC recurrence rate in non-NASH patients. However, a recent review compiling multiple observational studies reported that while, in fact, early studies warned of a higher HCC recurrence rate in HCV-related HCC patients, there is actually no significant change in recurrence linked to DAA treatment (36). We performed an unreported subanalysis of our own patient cohort removing patients diagnosed in the years 2012–2014 (prior to the widespread use of DAAs) which showed similar results, but all of which were underpowered. Another limitation of our study is the potential bias due to timing of HCC recurrence detection. The median survival post-recurrence will have some bias based on when the diagnosis is made which we could not account for given the dataset. Also, while most HCC recurrence post-transplant occur within 2 years, another limitation of the study is the relatively short median follow up at 3.4 years, which may miss late HCC recurrence. Additionally, the number of recurrences is relatively small leading to potential for bias in our subanalysis of overall survival. Unfortunately, we also did not have access to all the data surrounding reason for death within the database. However, of the available data, 72% of non-NASH and 69% of NASH deaths after recurrence were recorded as being secondary to malignancy with the remainder of causes of death being <10% for both cohorts except in the “Other” category. Additionally, many patients with recurrence decline and have a different reported ultimate cause of death despite the decline resulting from the recurrence. Finally, follow-up time for non-NASH patients in our study was 1,366 days (versus 924 for NASH patients). Unreported subanalysis was performed to remove non-NASH patients with longer follow-up and results were similar, but, again, underpowered.

Currently, increased early detection of HCC and surgical treatment offers the best therapeutic opportunity for HCC patients with any etiology (37). This study highlights, however, that differences do exist within the heterogeneous HCC patient population. These differences, likely linked to underlying etiology-specific tumor biology, should be the focus of future investigations to elucidate how we can exploit them and directly improve HCC outcomes.

## Data Availability

The original contributions presented in the study are included in the article/Supplementary Material, further inquiries can be directed to the corresponding author.

## Supplementary Material

Supplementary Figure S1 The propensity score match. **(A)** demonstrates covariates selected for use in the propensity score model. **(B)** demonstrates bias across each covariate between non-NASH and NASH recipients. These were compared before and after matching. On the bottom two graphs **(C)**, propensity score distribution from NASH (top) and non-NASH (bottom) populations are compared before [left, **(Ci)**] and after [right, **(Cii**)] matching.

## Abbreviations

AFP, alpha feto-protein; BMI, body mass index; CIT, cold ischemia time; CNS, central nervous system; CVA, cerebrovascular accident; DAA, direct-acting antiviral; DCD, donation after cardiac death; ESLD, end-stage liver disease; EtOH, alcohol; HBV, hepatitis B virus; HCC, hepatocellular carcinoma; HCV, hepatitis C virus; ICU, intensive care unit; LDRI, liver donor risk index; MELD, model for end stage liver disease; NAFLD, non-alcoholic fatty liver disease; NASH, non-alcoholic steatohepatitis; OPTN, Organ Procurement and Transplantation Network; PSM, propensity score matched; PVT, portal vein thrombosis; TACE, trans-arterial chemoembolization; TARE, trans-arterial radioembolization; UNOS, United Network for Organ Sharing.
