# Supplementary figures and images for: Reduced Rates of Post-Transplant Recurrent Hepatocellular Carcinoma in Non-Alcoholic Steatohepatitis: A Propensity Score Matched Analysis
Source: Transpl Int. 2022 Jul 5;35:10175. doi: 10.3389/ti.2022.10175 (PMC9294152; doi:10.3389/ti.2022.10175)

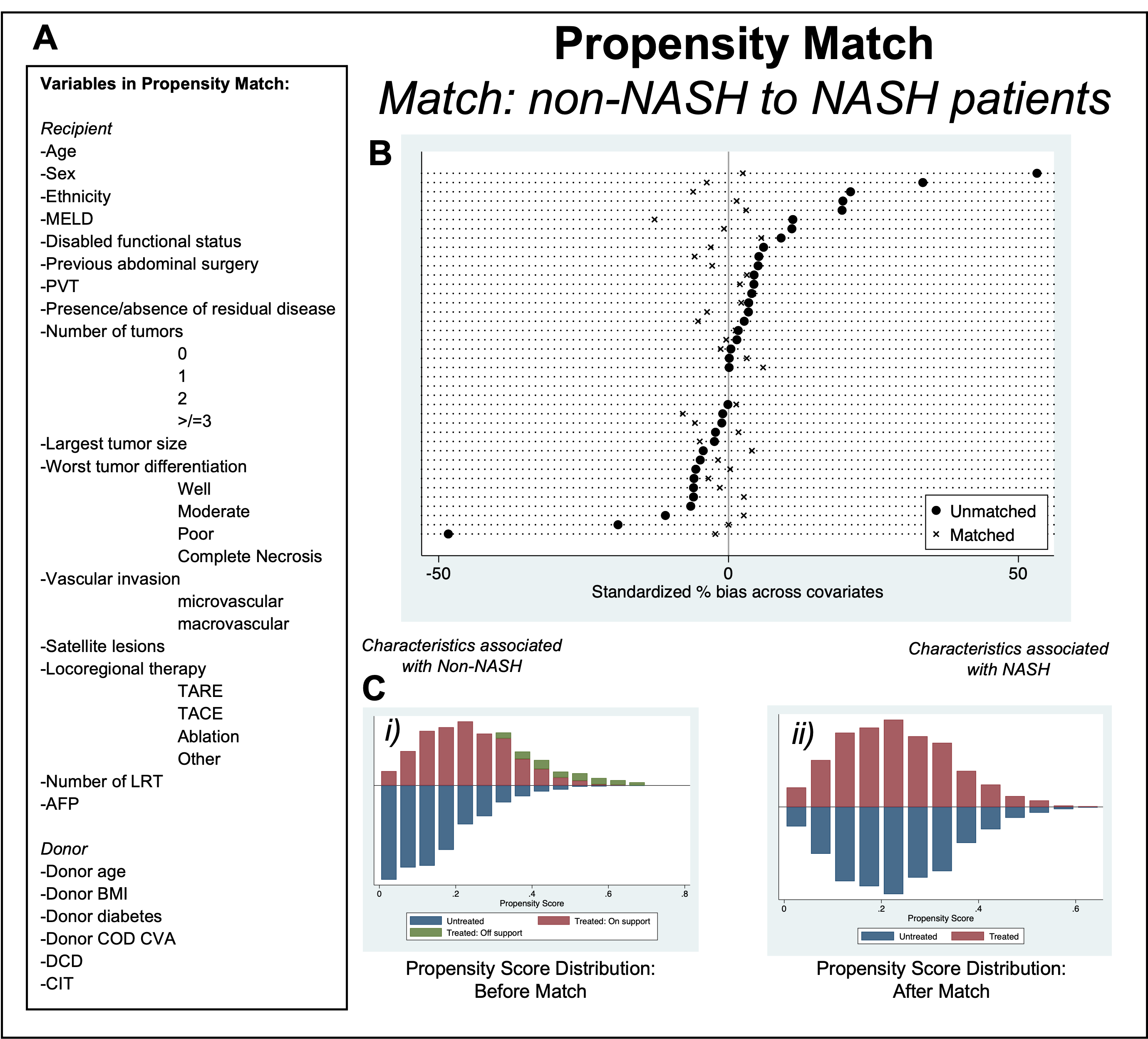

Supplement: Supplementary file 1 [file Image1.png]
