# Supplementary material for: Reduced Rates of Post-Transplant Recurrent Hepatocellular Carcinoma in Non-Alcoholic Steatohepatitis: A Propensity Score Matched Analysis
Source: Transpl Int. 2022 Jul 5;35:10175. doi: 10.3389/ti.2022.10175 (PMC9294152; doi:10.3389/ti.2022.10175)
Supplement: Supplementary file 2 [file DataSheet1.docx]

| **Table S1:** Unmatched baseline characteristics between NASH and non-NASH recipients with HCC | | | | | |  | | |  |
| --- | --- | --- | --- | --- | --- | --- | --- | --- | --- |
|  | **Unmatched** | | |  |  |  |  |  |  |
|  | NASH | Non-NASH | p-value |  |  |  |  |  |  |
| Number | 1,405 | 6,086 |  |  |  |  |  |  |  |
| Median followup (days) | 924 (365-1707) | 1,366 (678-1,898) |  |  |  |  |  |  |  |
|  |  |  |  |  |  |  |  |  |  |
| *Recipient characteristics* |  |  |  |  |  |  |  |  |  |
| Age | 65 (60-68) | 61 (57-65) | <0.01 |  |  |  |  |  |  |
| Female sex | 504 (35.87%) | 1,249 (20.52%) | <0.01 |  |  |  |  |  |  |
| Ethnicity |  |  | <0.01 |  |  |  |  |  |  |
| White | 1,050 (74.73%) | 3,935 (64.66%) |  |  |  |  |  |  |  |
| Black | 14 (1.00%) | 737 (12.11%) |  |  |  |  |  |  |  |
| Other | 341 (24.27%) | 1,414 (23.23% |  |  |  |  |  |  |  |
| BMI | 31.71 (28.08-35.48) | 27.93 (24.88-31.71) | <0.01 |  |  |  |  |  |  |
| Pre-exception MELD | 12 (9-16) | 11 (8-15) | <0.01 |  |  |  |  |  |  |
| AFP |  |  | <0.01 |  |  |  |  |  |  |
| <100 ng/mL | 1,187 (93.76%) | 5,041 (87.65%) |  |  |  |  |  |  |  |
| 100-399 ng/mL | 63 (4.98%) | 533 (9.27%) |  |  |  |  |  |  |  |
| ≥400 ng/mL | 16 (1.26%) | 177 (3.08%) |  |  |  |  |  |  |  |
| Locoregional therapy |  |  |  |  |  |  |  |  |  |
| TACE | 863 (61.42%) | 3,875 (63.67%) | 0.12 |  |  |  |  |  |  |
| TARE | 170 (12.10%) | 626 (10.29%) | 0.05 |  |  |  |  |  |  |
| Ablation | 445 (31.67%) | 2,018 (33.16%) | 0.30 |  |  |  |  |  |  |
| Other | 17 (1.21%) | 35 (0.58%) | 0.02 |  |  |  |  |  |  |
| Number of locoregional treatments | |  | 0.35 |  |  |  |  |  |  |
| 0 | 181 (12.88%) | 796 (13.08%) |  |  |  |  |  |  |  |
| 1 | 852 (60.64%) | 3,589 (58.97%) |  |  |  |  |  |  |  |
| 2 | 298 (21.21%) | 1,306 (21.46%) |  |  |  |  |  |  |  |
| ≥3 | 74 (5.27%) | 395 (6.49%) |  |  |  |  |  |  |  |
| Disabled functional status | 203 (14.45%) | 845 (13.88%) | 0.58 |  |  |  |  |  |  |
| Diabetes mellitus | 981 (71.66%) | 1,644 (27.34%) | <0.01 |  |  |  |  |  |  |
| Portal vein thrombosis | 230 (16.39%) | 775 (12.77%) | <0.01 |  |  |  |  |  |  |
| Hemodialysis | 10 (0.71%) | 80 (1.31%) | 0.08 |  |  |  |  |  |  |
| Previous abdominal surgery | 760 (54.09%) | 2,766 (45.45%) | <0.01 |  |  |  |  |  |  |
| Multiorgan | 27 (1.92%) | 117 (1.92%) | 0.99 |  |  |  |  |  |  |
| Primary diagnosis |  |  | - |  |  |  |  |  |  |
| NASH | 1,405 (100.00%) | 0 (0.0%) |  |  |  |  |  |  |  |
| HCV | 0 (0.0%) | 3,996 (66.44%) |  |  |  |  |  |  |  |
| HBV | 0 (0.0%) | 381 (6.34%) |  |  |  |  |  |  |  |
| EtOH | 0 (0.0%) | 1,281 (21.30%) |  |  |  |  |  |  |  |
| Other* | 0 (0.0%) | 356 (5.92%) |  |  |  |  |  |  |  |
|  |  |  |  |  |  |  |  |  |  |
| *Donor characteristics* |  |  |  |  |  |  |  |  |  |
| Age | 46 (31-59) | 43 (28-55) | <0.01 |  |  |  |  |  |  |
| Female sex | 589 (41.92%) | 2,432 (39.96%) | 0.18 |  |  |  |  |  |  |
| BMI | 27.62 (23.81-32.54) | 27.14 (23.62-31.31) | <0.01 |  |  |  |  |  |  |
| Diabetes mellitus | 203 (14.45%) | 784 (12.88%) | 0.13 |  |  |  |  |  |  |
| Macrosteatosis (%) | 5 (0-10) | 5 (0-10) | 0.06 |  |  |  |  |  |  |
| Inotrope support | 664 (47.26%) | 2,797 (45.96%) | 0.39 |  |  |  |  |  |  |
| LDRI | 1.61 (1.29-1.93) | 1.54 (1.25-1.88) | <0.01 |  |  |  |  |  |  |
| Cause of death |  |  | 0.81 |  |  |  |  |  |  |
| Anoxia | 515 (36.65%) | 2,280 (37.46%) |  |  |  |  |  |  |  |
| CVA | 468 (33.31%) | 1,972 (32.40%) |  |  |  |  |  |  |  |
| Head trauma | 390 (27.76%) | 1,694 (27.83%) |  |  |  |  |  |  |  |
| CNS tumor | 9 (0.64%) | 28 (0.46%) |  |  |  |  |  |  |  |
| Other | 23 (1.64%) | 112 (1.84%) |  |  |  |  |  |  |  |
| DCD | 113 (8.04%) | 495 (8.13%) | 0.96 |  |  |  |  |  |  |
|  |  |  |  |  |  |  |  |  |  |
| *Transplant details* |  |  |  |  |  |  |  |  |  |
| CIT (hours) | 5.80 (4.53-7.17) | 5.95 (4.63-7.40) | 0.01 |  |  |  |  |  |  |
| *Values are listed as number (percentage) or median +/- interquartile range unless otherwise stated*  *BMI: body mass index, NASH: non-alcoholic steatohepatitis, AFP: alpha fetoprotein, TACE: transarterial chemoembolization, TARE: transarterial radioembolization, HCV: Hepatitis C Virus, EtOH: alcohol, CVA: cerebrovascular accident, LDRI: Liver Donor Risk Index, CNS: central nervous system, DCD: donation after cardiac death, CIT: cold ischemia time* | | | | |  |  |  |  |  |
|  |  |  |  |  |  | | | | |
|  |  |  |  |  |  | | | | |
| **Includes metabolic, autoimmune and cholestatic diseases* | | | |  | | |  |  |  |

| **Table S2:** Tumor characteristics in unmatched transplant hepatectomy specimens | | | |
| --- | --- | --- | --- |
|  | **Unmatched** | | |
|  | NASH | Non-NASH | p-value |
| Number | 1,405 | 6,086 |  |
|  |  |  |  |
| No tumor on explant | 95 (6.76%) | 352 (5.78%) | 0.17 |
| Number of tumors |  |  | 0.10 |
| 1 | 635 (45.20%) | 2,943 (48.36%) |  |
| 2 | 327 (23.27%) | 1,278 (21.00%) |  |
| 3 | 149 (10.60%) | 690 (11.34%) |  |
| ≥4 | 199 (14.16%) | 823 (13.52%) |  |
| Largest tumor size (cm) | 2.5 (1.5-3.5) | 2.5 (1.5-3.5) | 0.99 |
| Tumor differentiation* |  |  | 0.37 |
| Complete necrosis | 362 (25.77%) | 1,677 (27.56%) |  |
| Well | 311 (22.14%) | 1,329 (21.84%) |  |
| Moderate | 649 (46.19%) | 2,683 (44.08%) |  |
| Poor | 83 (5.91%) | 397 (6.52%) |  |
| Vascular invasion |  |  | 0.54 |
| Microvascular | 153 (10.89%) | 715 (11.75%) |  |
| Macrovascular | 23 (1.64%) | 86 (1.41%) |  |
| Satellite lesions | 75 (5.34%) | 299 (4.91%) | 0.50 |

Values are listed as number (percentage) or median +/- interquartile range unless otherwise stated

*Differentiation of worst tumor

| **Table S3:** Unmatched transplant outcomes by diagnosis of NASH | | | | | |  |  |
| --- | --- | --- | --- | --- | --- | --- | --- |
|  | NASH | Non-NASH | HR/SHR | 95% CI | p-value |  |  |
| Number | 1,405 | 6,086 |  |  |  |  |  |
|  |  |  |  |  |  |  |  |
| Acute Rejection within 1 year | 80 (8.32%) | 335 (7.72%) | - | - | 0.55 |  |  |
|  |  |  |  |  |  |  |  |
| *Recurrent Malignancy* | |  | (SHR) |  |  |  |  |
| 5-year | 5.61% | 8.27% | 0.65 | 0.48-0.89 | 0.01 |  |  |
| Median time to recurrence* | 426 (213-930) | 427 (217-790) | - | - | 0.86 |  |  |
|  |  |  |  |  |  |  |  |
| *Post-transplant survival* | |  | (HR) |  |  |  |  |
| Overall | - | - | 0.99 | 0.85-1.16 | 0.94 |  |  |
| 1-year | 93.10% | 94.27% | - | - | 0.11 |  |  |
| 3-year | 86.16% | 85.41% | - | - | 0.97 |  |  |
| 5-year | 79.87% | 79.13% | - | - | 0.86 |  |  |

*Values are listed as percent, number (percentage) or median +/- interquartile range unless otherwise stated*

*For patients with recurrent HCC only

**Supplementary Figure S1**. The propensity score match. (A) demonstrates covariates selected for use in the propensity score model. (B) demonstrates bias across each covariate between non-NASH and NASH recipients. These were compared before and after matching. On the bottom two graphs (C), propensity score distribution from NASH (top) and non-NASH (bottom) populations are compared before (left, Ci) and after (right, Cii) matching.
